# External and internal microbiomes of Antarctic nematodes are distinct, but more similar to each other than the surrounding environment

**DOI:** 10.2478/jofnem-2023-0004

**Published:** 2023-03-09

**Authors:** J. Parr McQueen, K. Gattoni, E.M.S. Gendron, S.K. Schmidt, P. Sommers, D. L. Porazinska

**Affiliations:** Department of Entomology and Nematology, University of Florida, FL 32611 Florida USA; Department of Ecology and Evolutionary Biology, University of Colorado Boulder, CO 80309 Colorado Boulder USA

**Keywords:** Antarctica, bacteria, community assembly, ecology, *Eudorylaimus antarcticus*, metabarcoding, *Plectus murrayi*

## Abstract

Host-associated microbiomes have primarily been examined in the context of their internal microbial communities, but many animal species also contain microorganisms on external host surfaces that are important to host physiology. For nematodes, single strains of bacteria are known to adhere to the cuticle (e.g., *Pasteuria penetrans*), but the structure of a full external microbial community is uncertain. In prior research, we showed that internal gut microbiomes of nematodes (*Plectus murrayi*, *Eudorylaimus antarcticus*) and tardigrades from Antarctica’s McMurdo Dry Valleys were distinct from the surrounding environment and primarily driven by host identity. Building on this work, we extracted an additional set of individuals containing intact external microbiomes and amplified them for 16S and 18S rRNA metabarcoding. Our results showed that external bacterial microbiomes were more diverse than internal microbiomes, but less diverse than the surrounding environment. Host-specific bacterial compositional patterns were observed, and external microbiomes were most similar to their respective internal microbiomes. However, external microbiomes were more influenced by the environment than the internal microbiomes were. Non-host eukaryotic communities were similar in diversity to internal eukaryotic communities, but exhibited more stochastic patterns of assembly compared to bacterial communities, suggesting the lack of a structured external eukaryotic microbiome. Altogether, we provide evidence that nematode and tardigrade cuticles are inhabited by robust bacterial communities that are substantially influenced by the host, albeit less so than internal microbiomes are.

Recent investigation of host-associated microbiomes has resulted in a paradigm shift to no longer viewing animal species and their microorganisms as separate organisms, but instead as fully interdependent metaorganisms (Bosch and McFall-Ngai, 2011). Host-microbiome complexes have been studied across a wide range of animal species with significant microbial contribution to the metaorganism. Bacteria, archaea, viruses, fungi, and other microbial eukaryotes (e.g., protists) are the main components of animal microbiomes (Marchesi, 2010; Laforest-Lapointe and Arrieta, 2018). In humans, bacterial cells alone (38 trillion) outnumber human host cells (30 trillion), along with hundreds of billions of non-host eukaryotic cells (Sender et al., 2016). In addition to their sheer abundance, many microbiomes have positive effects on their animal hosts (Douglas, 2014; Hammer et al., 2019). For example, microorganisms inhabiting host guts have been shown to provide strong benefits to animal health (Peixoto et al., 2021), such as synthesizing essential amino acids in aphids (Douglas, 1996) or actively fighting infection in honey bees (Raymann and Moran, 2018). In humans, the gut microbiome has been linked to a wide range of functions, from cognitive development (Carlson et al., 2018) and behavior (Dinan et al., 2015) to dysbiosis (e.g., cancer, obesity, autism) (Thursby and Juge, 2017), illustrating the extent of possible ways microbiomes can influence their hosts.

The processes by which microorganisms assemble into communities, such as microbiomes, can be described by the ecological theory of community assembly (Nemergut et al., 2013) and can be placed into two generalized categories, defined as either stochastic or deterministic (Zhou and Ning, 2017). Stochastic/neutral processes (e.g., ecological drift, founder effects, and birth-death events) explain community composition based on random chance (Chave, 2004). By contrast, deterministic processes explain communities through niche based selection such as abiotic (e.g., pH and nutrient resources) and biotic (e.g., competition) factors (Hirzel and Le Lay, 2008) that act as selective filters. In animals with strong connections to their microbiomes, deterministic factors generated by host physical (e.g., stoma size), chemical (e.g., gut pH), or behavioral characteristics (e.g., diet preference) drive the assembly and composition of internal microbiomes (Hammer et al., 2019). Other animals with weak connections to their microbiomes often have stochastic, absent, or microbial communities similar in composition to the surrounding environment (Hammer et al., 2019).

While the animal metaorganism concept has been largely studied in the context of host internal microbiomes, external microbiomes that may be equal in importance to the metaorganism, have also been reported (Byrd et al., 2018). Like internal microbiomes, external microbiomes can directly benefit their hosts (e.g., protection against pathogens via production of antimicrobial compounds) (Chiller et al., 2001). Generally, both internal and external microbiomes consist of the same main types of microorganisms but are compositionally distinct through association with different host organs. Moreover, external microbiomes can be highly location-specific and dependent on specific features of the host body. For example, the microbiome of the human back is distinct from that of the forearm (Chiller et al., 2001; Grice and Segre 2011), as is the microbiome of the salamander’s dorsal side distinct from that of the ventral side (Bataille et al., 2016). Other animal species (e.g., finches) lack a body region distinction (i.e., microbiomes of finches’ neck and preen gland areas are all similar) (Engel et al., 2018). Compared to the strong deterministic influence of host identity in the assembly of internal microbiomes, external microbiomes are thought to be driven more by environmental factors. This is due to the constant exposure to their surroundings, making them therefore more stochastically driven in relation to the host, but this is not always the case. For example, external microbiomes of bats have been shown to be influenced by a combination of both factors (Lemieux-Labonté et al., 2016), ungulates (e.g., Artiodactyla and Perissodactyla) by host phylogeny (Ross et al., 2018), and salamanders by either factor (Prado-Irwin et al., 2017; Muletz Wolz et al., 2018; Bird et al., 2018). Overall, microbiomes are present on external host structures/organs and have a positive influence on a wide range of metazoans.

Although nematodes are the most abundant animals on Earth, remarkably little is known about their internal or external microbiomes, with a primary focus given to internal microbiomes of only a handful of nematode species (e.g., *Caenorhabditis elegans*) (Dirksen et al., 2016; Zhang et al., 2017). The internal bacterial microbiome of *C. elegans* has been shown to be primarily driven by host identity (Berg et al., 2016a; Dirksen et al., 2016) such that the same species from a wide range of geographic habitats have similar microbiomes. Moreover, populations of *C. elegans* retrieved from their natural environments are able to retain their wild microbiomes even when cultured on *Escherichia coli* (Dirksen et al., 2016), suggesting that the largest component of the bacterial community consists of the host-influenced microbiome rather than ingested bacterial food. The importance of host factors has also been observed in other nematode species. In Antarctic Dry Valley streams, for example, microbiome compositions of omnivorous *Eudorylaimus antarcticus* (currently under taxonomic revaluation) and bacterivorous *Plectus murrayi* were influenced more by the host’s identity than by environmental factors such as stream or the type of microbial mat that the nematodes were isolated from (McQueen et al., 2022). Despite evidence that the microbiomes of these two distinct nematodes were deterministically shaped by the host, this may not be true for the entire Nematoda phylum. For example, in extensive surveys of marine nematode internal microbiomes, host identity was not significant, giving credence to the role of stochasticity for some nematodes (Schuelke et al., 2018; Boscaro et al., 2022; Vafeiadou et al., 2022). Tardigrade microbiomes have been studied even less than those of nematodes, but initial studies suggest that similarly to terrestrial nematodes, they might be driven by a combination of both host-specific and environmental factors (Vecchi et al., 2018; Kaczmarek et al., 2020; Mioduchowska et al., 2021; McQueen et al., 2022; Zawierucha et al., 2022).

Although much is known about the presence of specific bacterial strains adhering to nematode cuticles (e.g., *Pasteuria penetrans*) (Chen and Dickson, 1998), knowledge of entire external microbiome communities is limited. These external communities may protect the nematode against pathogenic fungi or bacteria as in humans, but there is little evidence to support the presence or functional significance of external nematode microbiomes. Part of the problem lies in methods that may not distinguish between internal and external microbiomes for animals such as nematodes. External microbiomes of macrometazoans (e.g., humans, birds, and fish) have been studied by collecting samples through swabbing, scraping, or placing and then removing sticky tape (Ross et al., 2019). Similar approaches for micrometazoans such as nematodes are not feasible due to the small size of the host body (<100 μm in width) (Brady and Weil, 2014), and, consequently, the internal and external microbiomes can be confounded. For example, strong host-microbiome interactions have been observed for *C. elegans* and *Pristionchus pacificus*, but the conclusions were unable to distinguish between external-internal microbial complexes (Rae et al., 2008). Similarly, although external microbiomes of plant parasitic *Meloidogyne incognita* and *Pratylenchus sp*. nematode species have been described (Elhady et al., 2017), the inferences were based on assumptions that plant parasites lack internal microbiomes.

Another potential challenge to studying nematode external microbiomes is linked to the way nematodes are extracted from their environments such as soil. Most nematode extraction methods, including the Baermann funnel (Baermann, 1917), Whitehead tray (Whitehead and Hemming, 1965), sieving/sugar centrifugation (Jenkins, 1964), and Oostenbrink elutriation (Seinhorst, 1962), rely on the use of water to separate nematodes from soil particles. Washing over sieves (e.g., 500 μm mesh size) with excess water separates nematodes not only from soil debris, but also from microorganisms adherent to their cuticles. In fact, the process of moving nematodes and tardigrades through a series of liquid washes (e.g., sterile water or M9 buffer) has been used to remove cuticle-adherent bacteria in order to study the internal microbiome (Derycke et al., 2016; Berg et al., 2016a; Dirksen et al., 2016; Schuelke et al., 2018; Vecchi et al., 2018; Vafeiadou et al., 2022). The effectiveness of washing to remove cuticle bacteria has also been confirmed through electron microscopy (Derycke et al., 2016), smash and plate counts (Berg et al., 2016a), and fluorescence *in situ* hybridization (FISH) (Rivera et al., 2022).

Terrestrial nematodes live in a bacterial world, with topsoil containing over 10 million bacterial cells for every single nematode present (Chemnitz and Weigelt, 2015). Because nematodes and tardigrades continuously encounter this microbial abundance and diversity, a natural expectation would be that their cuticles contain a community of attached microbes that are yet to be discovered. Nematodes contain a non-cellular, collagen filled cuticle that is characterized by elaborate physical structures including ridges, annulations, and pores that could provide favorable space for microbial attachment. Indeed, strains of *Yersinia sp*. can form a biofilm on *C. elegans* nematodes in culture (Tan and Darby, 2004). Nematodes also actively disseminate bacteria through the soil matrix, but it is unclear if this spread occurs through defecation or from shedding of bacteria attached to the cuticle (Anderson et al., 2003). In comparison, tardigrades have 8 legs, each terminating in four to six claws, providing even more surficial space for microbes (Schill 2018; Czerneková and Vinopal 2021). Through scanning electron microscopy (SEM) and FISH, bacteria have been observed on the cuticle of nematodes and tardigrades (Bellec et al., 2018; Guidetti et al., 2019), but it is unclear if this represents a cohesive and functioning community or random microorganisms. Overall, there is a need for a natural system in which nematodes and tardigrades can be extracted in a way that allows for the potential external microbiome to be retained and studied.

The McMurdo Dry Valleys of Antarctica are the coldest, windiest, and driest deserts on Earth (Doran, 2002). The landscape is dominated by gravely unvegetated soils and ephemeral streams that flow for 8-12 weeks during austral summers, when temperatures rise high enough to melt adjacent glaciers (Fountain et al., 1999). Within the streams, there are different types of cyanobacterial mats with microbial communities that act as the habitat and food source for microinvertebrates including nematodes, tardigrades, and rotifers. Based on the dominance of specific cyanobacterial species, mats can be visually differentiated by their colors (Mcknight et al., 1999). While black mats are dominated by *Nostoc* and grow within stream margins, orange mats are characterized by a higher prevalence of Oscillatoriales and are typically found within the central flow of streams. Within both mat types there is a limited but well-characterized community of microinvertebrates consisting of 2 morphologically distinct nematodes (*Eudorylaimus antarcticus* and *Plectus murrayi*), 2-3 tardigrade and 15 rotifer species (Treonis et al., 1999; Porazinska et al., 2002; Adams et al., 2006). C and N stable isotope signatures have demonstrated that this simplified natural ecosystem functions at three distinct trophic levels of biotic interactions (Shaw et al., 2018). Photosynthesizing Cyanobacteria (black and orange), along with heterotrophic microbial communities, form the base of the food web. One of the nematode species, *P. murrayi*, as well as tardigrades and rotifers, most likely feed on bacteria and hence are thought to occupy the second (bacterial-feeding) trophic level. The second nematode species, *E. antarcticus*, is the sole occupant of the third and apex trophic level. Based on the C and N isotope ratios, it is thought to be the lone omnivore/predator in this system feeding on both the microbial mat communities and the other microinvertebrates (Shaw et al., 2018), although earlier studies proposed *E. antarcticus* to be an algivore (Wall, 2007) or an omnivore like other species of this genus (Yeates et al., 1993; Hodda, 2022). Vascular plants and aboveground macrometazoans are not present in the Dry Valleys.

The goal of our study was to analyze the external microbiome of nematodes and tardigrades by using methods that avoid the pitfalls of previously discussed extraction methods. Here, we demonstrate that nematodes and tardigrades can be isolated from microbial mats by directly picking individual specimens (e.g., with a metal or eyelash pick) from mats placed in sterile Petri dishes under a dissecting scope. Direct picking retains cuticle-adherent microorganisms that would otherwise be washed off with traditional washing and sieving steps. Although directly picked nematodes contain both external and internal microbiomes, by moving half of the specimens through a series of sterile washes, it is possible to study external microbiomes by bioinformatically subtracting sequencing reads of washed specimens from those that were unwashed. We hypothesized that nematode and tardigrade external microbiomes would be distinct from their internal microbiomes, but also from adjacent microbial mats. Furthermore, we hypothesized that due to the physical location and fewer filtering mechanisms (i.e., not passing through the stoma), external microbiome assembly would be more driven by environmental factors and hence share more similarity with mats than with internal microbiomes, which are known to be distinct from mats and host-specific.

## Materials and Methods

### Sample collection and DNA processing

Full site descriptions are provided elsewhere (McQueen et al., 2022), but in brief, two types of cyanobacterial mats (black and orange) were collected during 18-21 January 2019 from four seasonally active streams (Canada, Bowles Creek, Delta, Von Guerard) in Taylor Valley, Antarctica. For each stream, one representative plot with both mat types (2 m in radius) was randomly selected, and three replicates of each mat type without any sediments (7 cm x 7 cm) were collected with a sterile scalpel for a total of 24 mat samples (12 black and 12 orange). Samples were frozen, kept in the dark, transported to the University of Florida, and stored at -20°C. Approximately 30 g of mat samples were slowly defrosted (10°C every 24 hours) to 4°C and examined for microinvertebrates in sterile Petri dishes under a dissecting microscope. No decanting, sieving, or disturbance of any kind was performed to keep all possible adherent external microbiomes intact. Single specimens visually identified as alive *E. antarcticus* (n=128) (currently undergoing taxonomic revision), *P. murrayi* (n=345), and Tardigrada (n=169) were manually picked from mats directly with a metal pick, and approximately half were vigorously moved through three washes of cold sterile water (*E. antarcticus* n=88, *P. murrayi* n=176, Tardigrada n=94), while the other half remained unwashed (*E. antarcticus* n=40, *P. murrayi* n=169, Tardigrada n=75). To validate the effectiveness of washing in the removal of external microorganisms, we compared washed and unwashed specimens using SEM at the University of Florida Interdisciplinary Center for Biotechnology Research (details below). Following isolation, single nematode or tardigrade specimens were placed in individual microcentrifuge tubes and DNA extracted using 25 μL of a proteinase K lysis buffer (McQueen et al., 2022). Substrate mat DNA was extracted from ~0.3 g of mat slurry with a Qiagen DNeasy PowerSoil Kit. Although extracted mat DNA could theoretically also contain DNA from microinvertebrates and their microbiomes, due to their general low abundance in mats, the likelihood of recovering even a single individual in the ~0.3 g was very low. High throughput metabarcoding was used to characterize bacterial and eukaryotic microbial communities with 16S (515F/926R) and 18S (1391f/EukBr) rRNA gene markers (Caporaso et al., 2012) following the Earth Microbiome Project protocols (https://earthmicrobiome.org/) To confirm successful amplification, all PCR-ed samples and technical replicates were visualized using gel electrophoresis (1.5% agarose). Three PCR replicates were pooled and sent to the Hubbard Center for Genome Studies, University of New Hampshire, along with PCR and lysis buffer negative controls for barcode attachment, library preparation, and paired-end sequencing on an Illumina HiSeq 2500 (2x250bp) (Illumina Inc., CA, USA). Due to the limited number of available barcodes, samples were split among four separate sequencing runs. To examine potential variation among the runs, DNA from the same 16 samples were sequenced on two of the four runs.

## Bioinformatics and Construction of External Microbiomes

Following read demultiplexing by the sequencing facility, reads were imported into QIIME2 (Bolyen et al., 2019) and primers were removed with cutadapt (Martin, 2011). Initial data screening included trimming reads of nucleotides at < 30 quality score, as well as unpaired forward 16S reads (196 bp) and paired 18S reads (average 131 bp) being de-novo clustered to 100% similarity amplicon sequence variants (ASVs) using DADA2 (Callahan et al., 2016). Filtering criteria during the DADA2 processing included input quality filtering, chimera removal, and an algorithmic error model of the sequencing instrument. Taxonomy was assigned using the assign_taxonomy.py script in QIIME1.9 (Caporaso et al., 2010) against the SILVA 138 database for 16S reads and the SILVA 111 database for 18S reads (Quast et al., 2013), both with any “uncultured” reference sequences removed. From the constructed table, non-bacterial sequences in the 16S ASV table and non-eukaryotic sequences in the 18S ASV table were removed. In addition, 18S sequences with hits to a specific microinvertebrate host were removed only from that host’s microbial community, as were ASVs with poor assignments (<90% of query coverage or <95% ID) removed from all hosts. If present, any of the few sequences identified in negative controls were subtracted from experimental samples. Reads assigned to *Phormidesmis* were changed to *Phormidium* due to the uncertain nature of this cyanobacterial clade (Komárek et al., 2009; Thomazeau et al., 2010; Raabová et al., 2019). Based on ASV rarefaction curves indicating insufficient sequencing depth to recover the full diversity, 184 samples with less than 100 total 16S reads or 18S reads were discarded.

**Figure 1:**
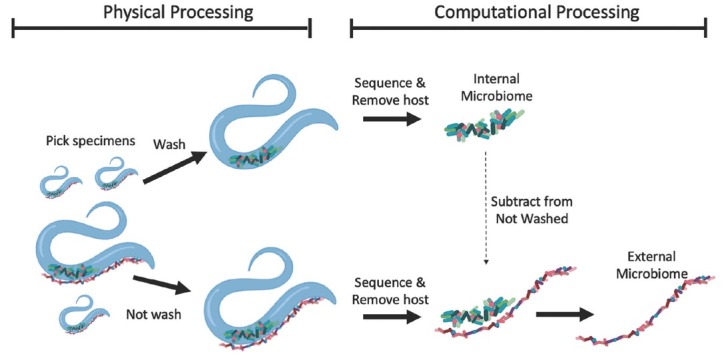
Graphical abstract of methods used to construct external and internal microbiomes of nematodes and tardigrades. For internal microbiomes, microinvertebrates were washed, sequenced, and host sequences subtracted before being averaged for each of the 24 mat replicates and 3 host types. For external microbiomes, unwashed microinvertebrates were sequenced, host sequences subtracted, and averaged for each of the 24 mat replicates and host types. The internal ASV abundances were then subtracted from the corresponding ASVs of unwashed community of the same mat replicate and microinvertebrate type to create the final external microbiome.

Across all samples, the total number of unwashed individuals of *E. antarcticus*, *P. murrayi*, and Tardigrada were 32, 103, and 72, respectively; the total number of washed individuals of *E. antarcticus*, *P. murrayi*, and Tardigrada were 52, 110, and 89, respectively. For each washed microinvertebrate individual, one representative internal microbiome (gut microbiome) was calculated by first removing all host ASVs and then averaging all remaining individuals for each of the 24 mat replicates. External microbiomes of all microinvertebrates were derived bioinformatically (Fig. 1) using the following steps. First, host ASVs were removed from all unwashed (and thus containing both external and internal microbiomes) individuals of microinvertebrates, and remaining ASVs were averaged to create one unwashed microbiome per each mat replicate. Second, to derive representative external microbiomes, ASVs (based on absolute abundance) of internal microbiomes of washed individuals were subtracted from the corresponding (i.e., assigning to the same ASV) ASVs of unwashed microbiomes for each mat replicate (Fig. 1). Although the number of reads within unwashed ASVs were generally higher than as those in the washed group, any subtractions resulting in negative values (i.e., reads were present in washed but not unwashed microbiomes) were equalized to zero. Although we refer to “microbiome” as the entire detected microbial community, we acknowledge that this may represent not only resident microorganisms but also non-viable eDNA.

## Statistics and Visualization

Statistics were performed in R Version 3.6.1 (R Core Team, 2020). Prior to any analyses, all ASV read counts were converted to relative abundance. Alpha diversity metrics (i.e., ASV Richness, Simpson’s, Shannon’s, and Faith’s Phylogenetic Diversity) were calculated with Hill Numbers using hill_taxa() from the *hillR* package (Alberdi and Gilbert, 2019). A linear model was run to compare alpha diversity of mat communities to microinvertebrates with the lm() function from the base R environment. For testing alpha diversity among microinvertebrate microbiomes, a linear mixed effects model was conducted with the lmer() function using Kenward-Roger degrees of freedom from the *lme4* package (Bates et al., 2015). Microinvertebrate host (*E. antarcticus* vs. *P. murrayi* vs. Tardigrada), microbiome type (external vs. internal), mat type (black vs. orange), and stream (Canada vs. Bowles Creek vs. Delta vs. Von Guerard) were tested as fixed factors with microinvertebrate nested under mat replicate as a random factor. Post hoc significance to compare groups was determined via the $contrasts output of emeans() from the *emmeans* package (Searle et al., 1980). Compositional differences among microbiomes based on Bray Curtis dissimilarity matrices were tested with the *vegan 2.5.7* package (Dixon 2003; Oksanen et al., 2019) through permutational analysis of variance (PERMANOVA) with 9999 permutations using the adonis() function. Three PERMANOVA tests were run; the first was to test the influence of microinvertebrate (*E. antarcticus* vs. *P. murrayi* vs. Tardigrada), microbiome type (external vs. internal), mat type (black vs. orange), and stream (Canada vs. Bowles Creek vs. Delta vs. Von Guerard) (independent variables) on all external and internal microbiome communities (dependent variables) together. The second was to test external microbiomes only (for the same above factors but excluding microbiome type), and the third was to test internal microbiomes only. Pair-wise, post hoc contrasts of any ordinated group centroids were calculated using the pairwise.adonis2() function within the *pairwiseAdonis* package (Martinez Arbizu, 2022). Bray Curtis dissimilarity matrices were also tested with the betadisper() function in *vegan* to analyze the multivariate homogeneity of group dispersions between each community. Values of dispersion were tested using the same linear mixed effects model as for microinvertebrate microbiomes alpha diversity metrics. Relative abundance of selected taxa (e.g., at phylum, family, and genus levels) was tested using a linear mixed effects model as described above. All figures were created in R using *ggplot2* (Wickham, 2016). Raw reads are available at the NCBI Sequence Read Archive with the project ID PRJNA851105. Documented code for the full bioinformatic pipeline, figure creation, and statistical analysis is available at www.WormsEtAl.com/externalmicrobiomes and https://github.com/WormsEtAl/ExternalMicrobiomes.The 16 duplicate samples sequenced on two different sequencing runs showed no differences in alpha diversity and community composition. In addition, the presented results below reflect community diversity and composition based on the relative abundance of microbial reads, and not of their absolute cell counts.

## Scanning Electron Imaging

To illustrate the effectiveness of the external microbiome removal via washing, ~60 additional specimens of *E. antarcticus* were picked from black mats from all four streams. Half of the individuals were passed through three washes of cold sterile water, while the other half remained unwashed. All specimens were then picked into a 4% glutaraldehyde fixative and transported to the ICBR where they were dehydrated with 2% osmium tetroxide and followed with EtOH replacement for critical point drying (Eisenback, 1986). Fixed and dried specimens were mounted on SEM stubs and sputter-coated with gold overnight. Samples were then imaged using a Hitachi SU5000 Schottky Field-Emission Variable Pressure microscope.

## Results

### Alpha Diversity Differences among Communities

**Figure 2:**
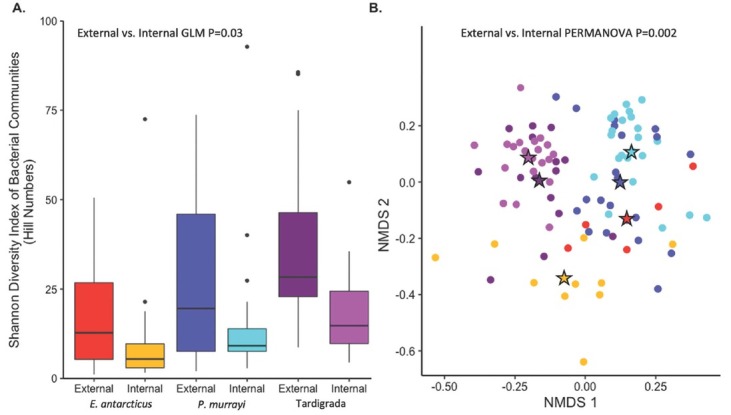
Diversity of microinvertebrate bacterial external and bacterial internal microbiomes. (A) Shannon’s diversity (box plot using Hill Numbers) with a significant difference between microbiomes (P=0.03, GLM) but not microinvertebrates (P=0.14), streams (P=0.22), or mat types (P=0.52). (B) Compositional difference based on Bray Curtis distance matrix visualized with a NMDS ordination, in which microinvertebrate host explained the most variation (R^2^ =0.14, PERMANOVA). Stars show centroid location of microbiome type, and solid circles show individual microbiomes.

All host bacterial microbiomes (external and internal) were significantly less diverse than mat microbiomes regardless of the alpha diversity metrics used (P<0.003, SI Table 1, SI Table 3). While neither mat type nor stream influenced alpha diversity of host microbiomes, (P>0.12, SI Table 2A), host ID and microbiome type were important factors (P<0.01). Specifically, microbiomes of Tardigrada were the most diverse (Richness and Faith’s PD), followed by *P. murrayi*, and *E. antarcticus* was the least diverse (Fig. 2A, SI Table 3). External microbiomes were more diverse (Shannon’s, Simpsons, and Faith’s PD) than the internal microbiomes (SI Table 2A), and trends were similar in direction and magnitude for all animal hosts (Fig. 2A, SI Table 2A, 3A). The difference between the two microbiome types (i.e., external vs internal) was lowest for *E. antarcticus*, followed by *P. murrayi*, and was highest for Tardigrada. In addition, the extent of variation (standard deviation) was higher for external than internal microbiomes across all microinvertebrate hosts (Fig. 2, SI Table 3A).

Similar to bacterial microbiomes, all host-associated eukaryotic microbiomes were less diverse than mat communities (SI Table 1). However, in contrast to bacterial microbiomes, there were no differences in diversity metrics between eukaryotic external and internal communities (SI Table 2B). There was also no effect of mat type nor stream (SI Table 3B), but external eukaryotic communities were significantly affected by the microinvertebrate host where the most diverse microbiomes were those of Tardigrada, followed by *P. murrayi* and then *E. antarcticus*.

### Compositional Differences among Communities

**SI Figure 1:**
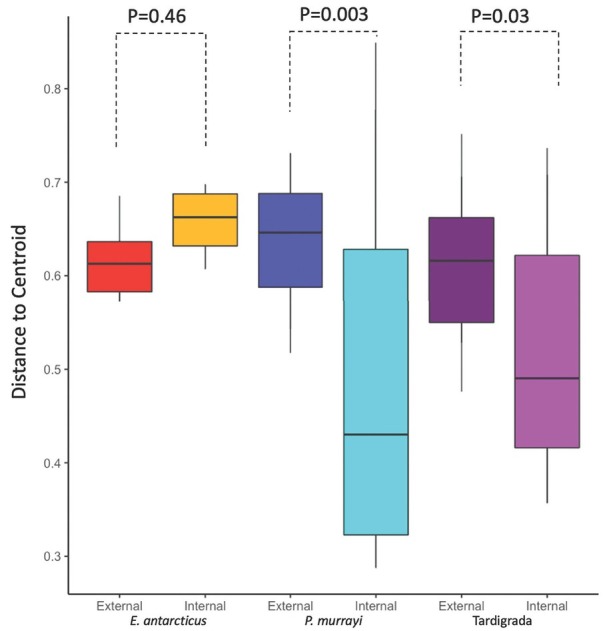
Dispersion values (a boxplot using distance to centroids based on Bray Curtis distance matrix) of external and internal bacterial microbiome composition for different hosts. In a mixed linear model, microinvertebrates did not significantly impact dispersion (P=0.44), but microbiome type did (P=0.03). Pairwise contrasts show that while external microbiomes of *P. murrayi* and Tardigrada are more variable than their internal microbiomes, *E. antarcticus* external and internal microbiomes are equally variable.

The composition of microinvertebrate bacterial communities was significantly affected by all tested factors including microinvertebrate host, microbiome type, mat type, and stream (PERMANOVA, P<0.01, Table 1A); however, the amount of variation explained by these factors varied greatly. Microinvertebrate host was the most important factor, explaining 14% of internal and external bacterial community variation (Table 1A), and separating microinvertebrate microbiomes into three distinct clusters in the NMDS space (Fig. 2B). Other factors were less significant, with stream explaining 6%, microbiome type 2%, and mat type 1% (Table 1A). Dispersion analysis indicated that both microbiome type (P=0.03) and stream (P<0.01) were significant, but not host (P=0.44) or mat type (P=0.64). Within microbiome type, specifically the variation among external bacterial microbiomes of *P. murrayi* and Tardigrada (but not of *E. antarcticus*) was significantly higher (P<0.03) than among internal microbiomes (SI Fig. 1). To better understand the contribution of specific factors driving each microbiome type, we subsequently examined their compositions separately (Table 1B,C). The composition of internal microbiomes was primarily influenced by microinvertebrate host (23% of variation) and to a lesser degree stream (7%), but not by mat type (P=0.23) (Table 1C). On the other hand, external microbiome composition was influenced by a combination of stream (10%) and microinvertebrate host (8%), followed by mat type (3%) (Table 1B). Splitting the models by microbiome types showed that while all factors tested were significant in explaining community composition, microinvertebrate host was the best predictor of internal microbiomes, while external microbiomes were more impacted by the environment, as stream explained the most variation in those cases.

**Table 1:** Differences in bacterial community composition of A. all microbiomes, B. external microbiomes, and C. internal microbiomes using a PERMANOVA.

	A. External and Internal Microbiomes		B. External Microbiomes		C. Internal Microbiomes	
	P	R^2^	P	R^2^	P	R^2^
Host	**< 0.00**	**0.14**	**< 0.00**	**0.08**	**< 0.00**	**0.23**
Microbiome	**< 0.00**	**0.02**	n.a.	n.a.	n.a.	n.a.
Mat	**< 0.00**	**0.01**	**0.02**	**0.03**	0.23	0.01
Stream	**< 0.00**	**0.06**	**< 0.00**	**0.10**	**< 0.00**	**0.07**
Host:Microbiome	**< 0.00**	**0.03**	n.a.	n.a.	n.a.	n.a.
Host:Mat	0.09	0.02	0.11	0.05	0.48	0.02
Microbiome:Mat	0.79	< 0.00	n.a.	n.a.	n.a.	n.a.
Host:Microbiome:Mat	0.47	0.01	n.a.	n.a.	n.a.	n.a.

Comparisons included microinvertebrate host (*E. antarcticus* or *P. murrayi* or tardigrades), microbiome type (external or internal), mat type (black or orange), stream (Canada or Bowles Creek or Delta or Von Guerard), and their interactions. The abbreviation “n.a.” is used to depict when a term was not included in the model. Factors explaining the largest variation (R^2^) of each model are underlined and statistically significant differences are highlighted in bold.

**SI Figure 2:**
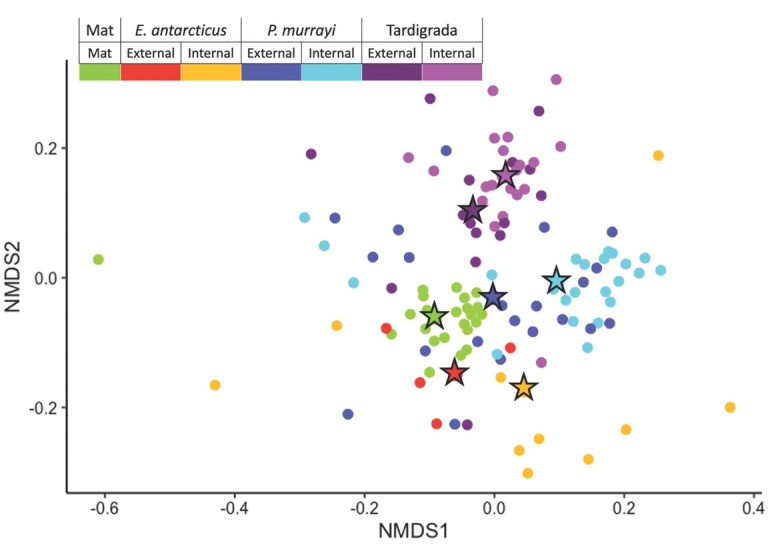
Compositional differences among bacterial microinvertebrate external and internal microbiomes as well as mats they were isolated from using Bray Curtis distance matrix visualized with a NMDS ordination. Circles indicate each community and stars show centroids of microbiome types for each animal host. All host microbiomes (internal and external) are distinct from mat communities (P<0.05), but external microbiomes are more similar to mats than internal microbiomes are to mats.

Despite significant differences in external and internal bacterial microbiomes (Table 1A), they largely overlapped in NDMS space (Fig. 2B), although the external microbiomes of all three microinvertebrates shifted to ordinate closer to mats than their internal microbiomes, as evident from the analysis of centroids (SI Fig. 2). This potentially higher mat-external microbiome similarity was further assessed with pairwise post hoc contrasts, showing that all microbiome types (external and internal of all animal types) were statistically distinct from mat microbiomes (P<0.002). However, the R^2^ values generated from pairwise contrasts comparing mats to external microbiomes (0.09 for *E. antarcticus*, 0.11 for *P. murrayi*, and 0.14 for Tardigrada) were uniformly lower than the R^2^ values comparing mats to internal communities (0.14 for *E. antarcticus*, 0.24 for *P. murrayi*, and 0.23 for Tardigrada), resulting in greater mat-external microbiome similarity compared to mat-internal microbiome differences.

**Figure 3:**
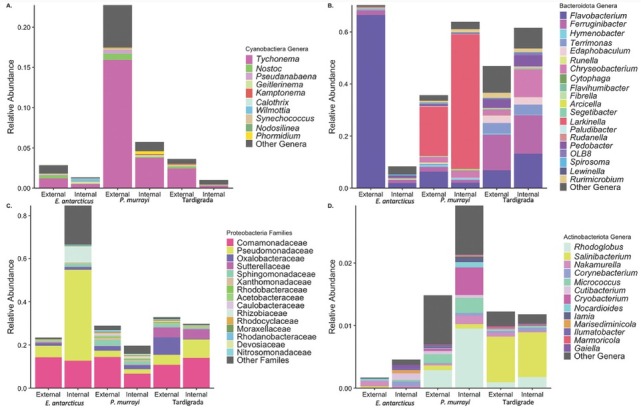
Relative abundance of bacterial communities of microinvertebrate external and internal microbiomes: (A) genera of Cyanobacteria, (B) genera of Bacteroidota, (C) families of Proteobacteria, (D) genera of Actinobacteriota.

The relative abundance of bacterial taxa within external and internal microbiomes differed across microinvertebrates (Fig. 3A-D, SI Table 5). All microbiomes were primarily comprised of Cyanobacteria, Proteobacteria, and Bacteroidota, totaling 87.0% of entire bacterial communities. In comparison to mats, both microbiome types were depleted in Cyanobacteria, however, more Cyanobacteria were present within the external microbiomes than in the internal microbiomes of all microinvertebrate hosts (P=0.01, Fig. 3A). *Tychonema* was the most abundant cyanobacterial genus within external and internal microbiomes of all microinvertebrates, comprising on average 9.5% and 3.0%, respectively. In contrast to the uniform trends of Cyanobacteria, patterns for Bacteroidota were more host-specific. For example, the relative abundance of Bacteroidota within external microbiomes of *E. antarcticus* was 66% higher than internal microbiomes (P< 0.01, Fig. 3B). This pattern was the opposite for *P. murrayi* and Tardigrada (P<0.001 and P=0.13, respectively). While *Tychonema* was the single dominant taxon within Cyanobacteria for all microinvertebrates, genera driving the overall abundance of Bacteroidota differed among microinvertebrates. *Flavobacterium* only dominated external microbiomes of *E. antarcticus* and was significantly more abundant compared to any other microbiome (P=0.01, pairwise post hoc contrasts). Both microbiomes of *P. murrayi* were dominated by *Larkinella*. Finally, both Tardigrada microbiomes were equally enriched in similar genera including *Ferruginibacter* and *Chryseobacterium*. Although there were no differences in the abundance of Proteobacteria between microbiome types (external or internal) (P=0.13, Fig. 3C) across all microinvertebrate hosts, external microbiomes of *E. antarcticus* were significantly more enriched in Proteobacteria compared to internal *E. antarcticus* microbiomes (P=0.01). Among Proteobacterial taxa, all microbiomes were equally dominated by the cryophilic *Polaromonas* in the family of Comamonadaceae (Fig. 3C). Except for the single genus *Pseudomonas* (in the family of Pseudomonadaceae) with significantly higher relative abundance within internal microbiomes of *E. antarcticus*, the abundance of other Proteobacterial taxa was mostly similar. The patterns for taxa within less abundant phyla (e.g., Actinobacteriota) were also highly host- and microbiome type-specific (Fig. 3D).

**Figure 4:**
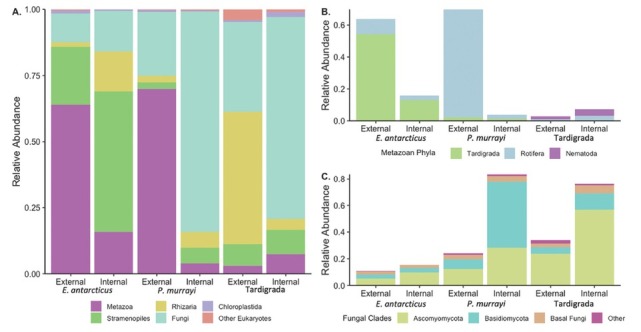
Relative abundance of eukaryotic communities of microinvertebrate external and internal microbiomes: (A) total non-host community (B) metazoan phyla, (C) fungal clades.

**SI Figure 3:**
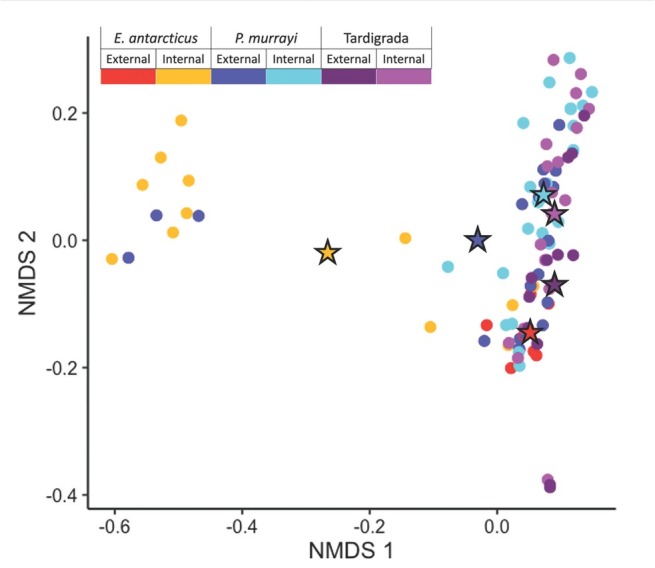
Compositional difference among eukaryotic microinvertebrate external and internal microbiomes, using Bray Curtis distance matrix visualized with a NMDS ordination. Circles indicate each community and stars centroid location of each microbiome type. Communities do not cluster by animal, microbiome type, mat type, or stream.

Although microinvertebrate host, microbiome type, and stream were significant for eukaryotic community composition, microbiomes did not visibly cluster by any factor (SI Fig. 3). The amount of community composition variation explained by each factor was substantially and consistently lower than for bacterial microbiomes (5% vs. 14%, 1.6% vs. 2.0%, and 3.4% vs. 6%, respectively) (Table 1A, SI Table 4A). Consequently, more community variation remained unexplained (79.6%), compared to bacterial communities (69%). When examining only external microbiomes, eukaryotes were equally explained by the host and stream (9%) (SI Table 4B). For only internal eukaryotic microbiomes, host explained the same amount of variation (9%), and the amount explained by stream lowered to 6% (SI Table 4C). Fungi made a significant contribution to all microbiomes (Figure 4A, SI Table 6) and its abundance significantly differed between microinvertebrate hosts (P<0.001), but not between microbiome type (P = 0.14, SI Table 5). Fungi (e.g., Ascomycota and Basidiomycota) were enriched in internal microbiomes of *P. murrayi* and Tardigrada, compared to all other communities with pairwise post hoc tests (P<0.0001, Fig. 4C). Generally, external microbiomes of nematode species were dominated by non-host metazoans, *E. antarcticus* by tardigrades, and *P. murrayi* by rotifers. In addition, internal microbiomes of *E. antarcticus* were characterized by the largest relative abundance (SI Table 6) of non-host metazoans including tardigrades and rotifers but no *P. murrayi* (Fig. 4B). No *E. antarcticus* was recovered in external microbiomes of *P. murrayi*, and no *P. murrayi* was in the external microbiomes of *E. antarcticus*.

### Verification of Washing Methods

**SI Figure 4:**
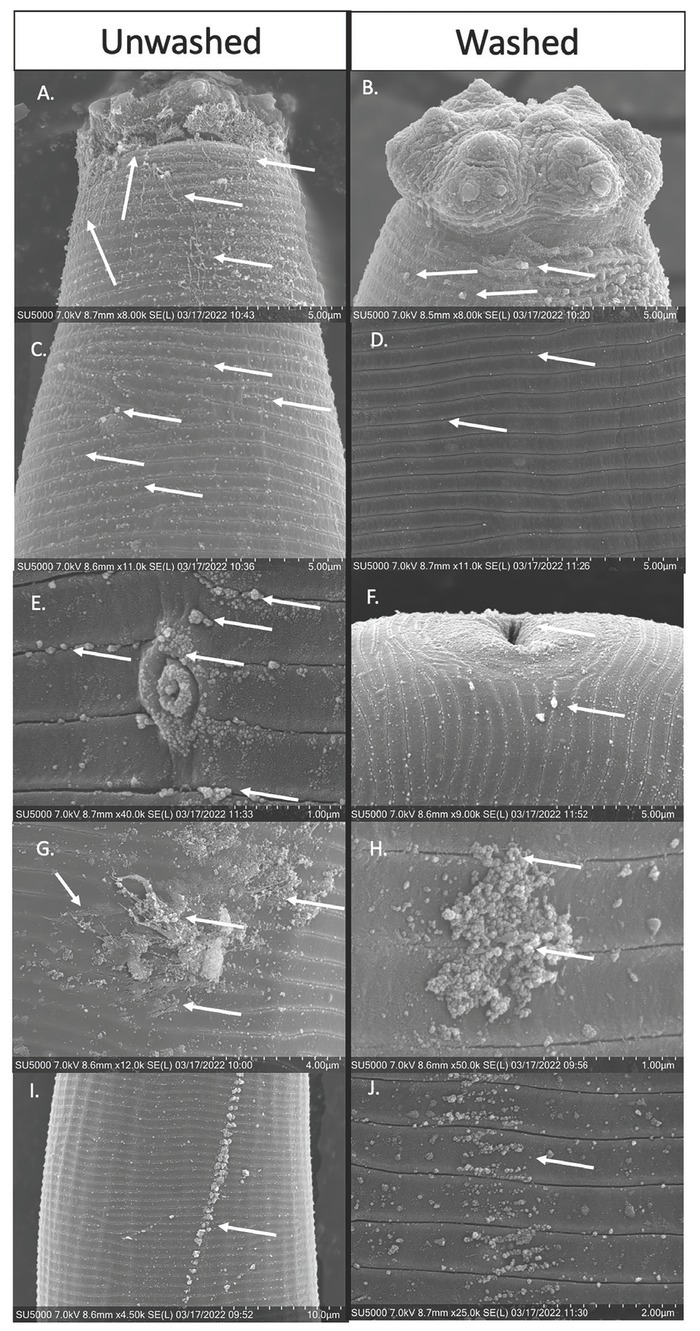
SEM images of either unwashed (left) or washed (right) *E. antarcticus* nematodes. A. Unwashed head region with arrows pointing to attached material and possible fungal hyphae. B. Washed head region with arrows pointing to the remaining attached material. C. Unwashed annules with arrows pointing to commonly attached foreign material. D. Washed annules with arrows pointing to remaining attached material. E. Unwashed somatic pore with arrows pointing to the common organic material. F. Washed vulva with an arrow pointing to remaining attached organic material. G. Unwashed cuticle with arrows showing a possible biofilm. H. Washed cuticle showing single attached cells indicated with arrows. I. Unwashed cuticle showing an off-axis line of attached material. J. Washed cuticle showing a similar off-axis line of material (as indicated with arrow) but reduced in quantity compared to the unwashed.

Specimens of washed and unwashed nematodes of both species were examined under SEM. Compared to the 9 washed *E. antarcticus* examined, all 6 unwashed individuals contained more adherent material along the entire length of the nematode body. Although washed nematodes did retain a small amount of this material, the great majority was consistently removed from all 9 individuals examined. Representative photos comparing different body regions of unwashed and washed individuals of *E. antarcticus* are provided in supplementary material (SI Fig. 4A-4J). Attached material was concentrated around nematode anatomical features such as the head (SI Fig. 4A,B), between annules and lateral lines (SI Fig. 4C,D), and around excretory pores (SI Fig. 4E). Organic material was most concentrated along the cervical region where the lips meet the body, as well as the stomatal opening. Sizes of organic materials varied considerably (0.05-1.3 μM) and likely consisted primarily of bacterial biofilm (SI Fig. 4G), clumps of mat fibers, and fungal hyphae (SI Fig. 4A). Single bacterial cells were also observed, but most were found under the possible biofilm-like layer (SI Fig. 4H). Interestingly, we routinely observed an off-axis line (i.e., not aligned to annulations or lateral field) of material in many of the unwashed specimens (SI Fig. 4I). Although remnants of this line were also present in the washed specimens, it was always less apparent (SI Fig. 4J). Overall, SEM demonstrated the effectiveness of nematode washing in removing the majority of potential external microbiomes.

## Discussion

Many animal species contain a collection of microorganisms that live in direct association with the host. This combined metaorganism has often been studied in the context of gut microbiomes, showing that microbiota provide direct functional benefits to nematodes (e.g., *C. elegans*) (Clark and Hodgkin, 2014; Berg et al., 2016b), and to many but not all metazoans (Hammer et al., 2019). Community assembly of a select few other nematode and tardigrade internal microbiomes have been described (Derycke et al., 2016; Berg et al., 2016a; Dirksen et al., 2016; Schuelke et al., 2018; Vecchi et al., 2018; McQueen et al., 2022; Vafeiadou et al., 2022), showing strong host influences on the internal microbial communities of terrestrial nematodes, which suggests a symbiotic importance as has been found in other hosts with deterministic microbiomes. In addition to gut microbiomes, host-associated microbiomes can also colonize the skin, and these external microbiomes likely hold equal importance to the host (Byrd et al., 2018; Ross et al., 2019). For nematodes and tardigrades, single strains of bacteria are known to adhere to the cuticle, but the community composition of the external microbial community has not been previously described.

Using nematodes and tardigrades from Antarctic cyanobacterial mats, we were able to computationally construct and describe the presence and composition of external microbiomes by comparing washed and unwashed specimens. For all microinvertebrate species (*E. antarcticus*, *P. murrayi*, and Tardigrada), external bacterial microbiomes were more diverse than their respective internal microbiomes, but less diverse than the surrounding microbial mats they inhabit and feed on, supporting our hypothesis that nematodes and tardigrades have diverse external microbiomes. This gradient of reduced diversity indicates greater deterministic selective pressure on internal microbiomes due to them potentially having more ecological “filters” (e.g., physical size limitation of the stoma aperture, behavioral feeding habits) and other unexplored factors influencing the community assembly (e.g., pH, feeding maceration) than external microbiomes. Due to the physical location of the external microbiome and its greater proximity to the environment, it would be expected that its composition would be less influenced by the host, with a greater influence of the environment. However, we found that bacterial composition of all host microbiomes was distinct from mats and microbiomes ordinated by host type with a complete overlap of external and internal microbiomes for each host. This indicates a larger role of the host on the external microbiome than we anticipated. Despite this, R^2^ values generated from pairwise contrasts comparing mats to external communities were lower for each host than R^2^ values comparing mats to respective internal communities. The lower explained variation indicates more overlap and similarity of the mats to the external microbiomes than of mats to internal microbiomes. Furthermore, a combined PERMANOVA model showed that although microbiome type (external or internal) was significant in explaining community composition, microinvertebrate host and stream were both more important in explaining community composition. Using the split model to examine external and internal microbiomes separately, the importance of environmental and host factors swapped, with external microbiomes being more explained by stream, while internal microbiomes were more explained by the microinvertebrate host, highlighting the larger influence the environment has on external microbiomes. This supports the first part of our hypothesis in that external microbiomes are distinct from mats and among microinvertebrates, but contrasts the second half, as there was a smaller difference between the composition of microbiome types (external or internal) than expected. Overall, external and internal microbiomes of a host were more similar to each other than they were different, with only 2% of variation explained by the microbiome type. However, our PERMANOVA was able to discern a greater influence of environmental factors for external microbiomes compared to internal microbiomes with external microbiomes more resembling mats. This can also be seen in relative abundance of communities, as external microbiomes of all animal types contained a higher relative abundance of Cyanobacteria than internal microbiomes, as they live in a substrate of cyanobacterial mats. This similarity of taxa between microbiomes, but reduction of diversity in the internal microbiome, may indicate that the cuticle provides a primary level of filtering for the assembly of the internal bacterial microbiome and acts as an important deterministic factor for the overall host-microbiome complex.

Although non-host eukaryotic microbiomes were less diverse than the eukaryotic community of the mats, there was no difference in the diversity between microbiome types (external or internal), nor did communities cluster by host or microbiome type. Instead, these communities appeared to follow a more stochastic assembly, which is reflected by the eukaryotic PERMANOVA model explaining 10.1% less of the community variation than for the bacterial communities. External and internal eukaryotic microbiomes were of the same diversity, and consistent patterns of taxa abundance were observed. There was also little similarity among shared feeding groups (i.e., *P. murrayi* and Tardigrada) or host phylum (i.e., *E. antarcticus* and *P. murrayi*). Although both nematode internal microbiomes were enriched in fungi compared to the external, these were comprised of distinct fungal communities (dominated by Ascomycota or Basidiomycota, respectively). In addition, the external microbiomes of both nematodes were dominated by non-host metazoans (e.g., rotifers and tardigrades), but this was not the case for the Tardigrada hosts. Unlike for fungi or bacteria, there is no SEM evidence suggesting that non-host metazoans have colonized the cuticle of nematodes and/or tardigrades, so instead these reads may originate from free-floating eDNA and indicate possible biotic interactions. *E. antarcticus* is well known as the sole omnivore/predator in the system (Shaw et al., 2018), so it is surprising that the external microbiome of *P. murrayi* contained similar levels of non-host metazoans. The dominance of non-host metazoans in nematode external microbiomes is even more intriguing given that we did observe ample fungi in SEM images, but sequencing data indicates ample non-host metazoan DNA. This opens the possibility for tardigrades and rotifers to be more physically connected to nematodes in day-to-day behavior and functions than previously thought.

External microbiomes hold the potential to act as a local species pool for internal gut microbiomes. Although nematode and tardigrade cuticles are impermeable to bacteria (Bird and Bird, 1991; Schill, 2018), transfer through the stoma is possible even for non-bacterial-feeding specialists including omnivorous nematodes. In our SEM imagery, we observed a greater concentration of organic matter on the lips of nematodes, even for the omnivorous *E. antarcticus*. Bacterivorous nematodes may actively feed by selecting specific bacterial species/strains for sustenance, but fortuitously draw in other bacteria previously attached to their own lips. We propose that the cuticle acts as an ecological filter to narrow down the regional pool that is further filtered to become the internal microbiome (the community most distinct from the mats). In support of this hypothesis, we found that the external microbiomes of all animal types were more diverse than their internal microbiomes, and that external and internal communities contained similar host-specific taxa. For example, *Larkinella* was found both in high concentrations of *P. murrayi* external and internal microbiomes, but not in other hosts. If *Larkinella* can colonize the *P. murrayi* cuticle, the internal microbiomes consequently might also contain high concentrations of *Larkinella* due to the constant exposure. In addition to indirect influences on assembly of what can attach to the cuticle, there may be direct host physiology influencing the external microbiome. In humans, for example, bacteria within the gut directly influences the composition of the skin microbiome (O’Neill et al., 2016), through the production and release of metabolites (Salem et al., 2018). Our results show a surprisingly large influence of host identity on external microbiomes, despite all hosts residing in the same environment. As the two nematodes are comprised of similar cuticle physical structure, there may be other host-specific interactions occurring. As part of the secretory and excretory system, pores exist in the cuticle of nematodes (Bird and Bird, 1991) and tardigrades (Schill, 2018; Czerneková and Vinopal, 2021) that produce secretions and other organic molecules. The functions of these compounds are mostly unknown, but in nematodes, these secretions produce a surface coat (i.e., glycocalyx) that is involved in protection against fungal pathogens (Blaxter et al., 1992). If nematodes secretions are actively mediating the attachment of bacteria to the cuticle, this could influence gut microbiome composition and vice versa.

As part of a normal lifecycle, nematodes move both vertically and horizontally throughout the soil matrix in search for food. Although soil matrix obscures observations of this movement, in food-depleted cultures, nematodes release signaling chemicals to activate movement around the Petri dish (Harvey, 2009; Kaplan et al., 2012). In addition to active transport, nematodes can be transported passively at local scales (meters) in the environment by earthworms and other meiofauna (Shapiro et al., 1993) and at regional scales by macrofauna (e.g., birds and large mammals), the wind, or water (Nkem et al., 2006; Frisch et al., 2007; Vanschoenwinkel et al., 2008; Ptatscheck et al., 2018). Regardless of the scale and transport, nematodes measurably contribute to the belowground ecosystem by spreading bacteria at a greater distance than any motile bacterium can by itself (Yang and van Elsas, 2018). While it remains unclear if this nematode-mediated spread is a result of defecation from the digestive tract or from external microorganisms detaching from the nematode cuticle (Anderson et al., 2003), by establishing the presence of diverse and distinct microbiomes, our study indicates that both scenarios could be equally important.

Although we were able to describe computationally generated external microbiomes, technical limitations currently prevent the direct analysis of external microbiomes. Due to the small body size of nematodes and tardigrades, we were unable to physically separate or sample only the external surface of the hosts. The major limitation of this issue is that we could not evaluate external microbiomes at the level of a single individual. Another inherent issue with the methods used for this study is the inability to distinguish potential cuticular microbiome differences associated with specific body locations as in other animals such as humans or salamanders (Chiller et al., 2001; Grice and Segre, 2011; Bataille et al., 2016). Because nematode cuticles vary along the nematode body, it could be expected that the microbiomes also vary. From our SEM images, we observed a greater concentration of organic matter around specific cuticular features (e.g., SE and amphidial pores, neck, and annulations), suggesting increased microbial abundance at these sites. Although the cuticles of *E. antarcticus* and *P. murrayi* are both annulated, cuticles of *P. murrayi* are well defined compared to the finely annulated *E. antarcticus* (Yeates, 1970). Consequently, gaps between annules of *P. murrayi* are wider and deeper and provide more space for attachment of external material than of *E. antarcticus*. In addition, caudal glands, exiting through a spinneret at the posterior end in *P. murrayi*, provide another inhabitable space for the external microbiome, all of which are absent in *E. antarcticus* (Yeates, 1970). Tardigrade cuticles contain larger folds along the body and legs, potentially trapping even more material (Schill, 2018). These physical body differences could explain the difference between external and internal microbiome diversity: the smallest for *E. antarcticus*, followed by *P. murrayi* and the largest for Tardigrada, but this could have been confounded with a similar pattern of internal diversity. Accounting for differences in raw diversity, dispersion analysis of communities showed that the external microbiomes of *P. murrayi* and Tardigrada were more variable than respective internal microbiomes, in contrast to the external and internal microbiomes of *E. antarcticus*, which were equally variable. Another limitation is the possibility of retaining adherent microorganisms in our internal microbiomes despite our washing steps. Although our SEM photos show most external microorganisms were removed, some did remain. More invasive washing steps, such as a bleach solution, are conceivable, but in practice are likely to damage the DNA of associated microorganisms including the internal microbiome. Although our insights focus on relative abundance, diversity, and composition, we are unable to evaluate the absolute abundance of microorganisms. More quantitative methods such as cell count and qPCR could be used in future studies to more precisely evaluate differences of abundance between communities. Overall, despite prevailing limitations, we were able to provide the most accurate representation of nematode and tardigrade external microbiomes to date.

In general, our study provides evidence that nematodes and tardigrades contain a robust bacterial external microbiome that is greater in diversity than the host’s internal microbiome. In addition, external bacterial microbiome composition was more influenced by environmental factors (i.e., stream, mat type) than internal microbiomes were, but both were most dependent on undescribed deterministic host factors. Eukaryotic external microbiomes appear to be less developed, with more stochastic patterns present. This could indicate a functional role of external bacterial microbiomes to the host, as animals who do not need a sustained external microbiome would not have evolved the behavioral or physical properties to influence that community.

## Figures and Tables

**SI Table 1:** A. Means and standard error of the mean (SE) of alpha diversity metrics (Shannon’s, Simpsons, Richness, Faith’s PD) for bacterial and eukaryotic mat communities. Alpha diversity metrics were calculated with Hill Numbers. B. Differences in alpha diversity comparing mats to host microinvertebrate microbiomes using a linear mixed model to test bacterial communities and then eukaryotic communities in a second linear mixed model.

A. Mat Community	Shannon’s Index		Simpsons Index		Richness		Faith’s PD	
	Mean	SE	Mean	SE	Mean	SE	Mean	SE
Bacteria	88.20	13.69	23.54	5.39	463.86	34.75	28.48	1.68
Eukaryota	8.72	1.80	4.29	0.73	73.67	8.73	13.51	0.86
**B. Mat vs. Hosts**	**Shannon’s Index**		**Simpsons Index**		**Richness**		**Faith’s PD**	
	**F**	**P**	**F**	**P**	**F**	**P**	**F**	**P**
Bacteria	44.02	**<0.01**	9.16	**<0.01**	25.81	**<0.01**	56.38	**<0.01**
Eukaryota	2.86	**0.09**	8.67	**<0.01**	78.04	**<0.01**	205.88	**<0.01**

Statistically significant differences are highlighted in bold.

**SI Table 2:** Differences in A. bacterial and B. eukaryotic host microbiome alpha diversity (Shannon’s, Simpsons, Richness, Faith’s PD) using a linear mixed model. Comparisons included microinvertebrate host (*E. antarcticus* or *P. murrayi* or Tardigrada), microbiome type (external or internal), mat type (black or orange), stream (Canada or Bowles Creek or Delta or Von Guerard), and their interactions. Alpha diversity metrics were based on ASVs and calculated with Hill Numbers.

A. Bacterial Diversity	Shannon’s Index		Simpsons Index		Richness		Faith’s PD	
	F	P	F	P	F	P	F	P
Host	2.04	0.15	0.54	0.59	**16.78**	**<0.00**	**18.01**	**<0.00**
Microbiome	**4.64**	**0.04**	**3.28**	**0.08**	2.07	0.16	**3.01**	**0.09**
Mat	0.42	0.52	0.31	0.58	0.97	0.33	0.46	0.50
Stream	1.60	0.22	2.23	0.12	1.13	0.37	1.09	0.38
Host:Microbiome	0.34	0.71	0.2	0.82	0.45	0.64	0.28	0.75
Host:Mat	0.26	0.78	0.27	0.76	2.11	0.14	2.11	0.14
Microbiome:Mat	0.22	0.64	0.86	0.36	0.25	0.62	0.06	0.81
Host:Microbiome:Mat	1.02	0.37	0.27	0.77	0.41	0.66	0.4	0.67
**B. Eukaryotic Diversity**	**Shannon’s Index**		**Simpsons Index**		**Richness**		**Faith’s PD**	
	**F**	**P**	**F**	**P**	**F**	**P**	**F**	**P**
Host	2.52	0.10	**3.52**	**0.04**	**2.70**	**0.08**	**3.88**	**0.03**
Microbiome	1.18	0.28	1.09	0.30	0.33	0.57	0.59	0.45
Mat	0.21	0.65	0.35	0.56	0.11	0.75	0.27	0.61
Stream	3.34	0.04	2.77	0.07	1.73	0.20	1.23	0.33
Host:Microbiome	0.26	0.77	0.27	0.77	0.44	0.64	0.85	0.44
Host:Mat	0.03	0.97	0.07	0.93	0.22	0.80	0.61	0.55
Microbiome:Mat	1.26	0.27	0.75	0.39	0.84	0.37	0.69	0.41
Host:Microbiome:Mat	0.00	1.00	0.33	0.72	0.65	0.53	0.53	0.60

Statistically significant differences are highlighted in bold.

**SI Table 3:** Means and standard error of the mean (SE) of alpha diversity metrics (Shannon’s, Simpsons, Richness, Faith’s PD) for A. bacterial and B. eukaryotic external and internal microbiomes of three hosts (*E. antarcticus* or *P. murrayi* or Tardigrada). Alpha diversity metrics were calculated with Hill Numbers.

A. Bacterial Diversity		Shannon’s Index		Simpsons Index		Richness		Faith’s PD	
		Mean	SE	Mean	SE	Mean	SE	Mean	SE
*E. antarcticus*	External	18.60	7.69	10.36	4.38	43.5	16.08	5.20	1.37
	Internal	12.46	5.76	8.35	3.83	24.92	9.51	3.47	0.99
*P. murrayi*	External	35.86	7.63	16.23	4.32	161.14	26.84	12.24	1.40
	Internal	22.25	7.38	9.21	2.71	114.09	25.58	9.75	1.39
Tardigrada	External	43.64	8.14	16.21	3.21	446.20	85.27	21.52	2.96
	Internal	22.64	4.96	8.99	1.70	297.30	56.03	15.84	2.12
**B. Eukaryotic Diversity**		**Shannon’s Index**		**Simpsons Index**		**Richness**		**Faith’s PD**	
		**Mean**	**SE**	**Mean**	**SE**	**Mean**	**SE**	**Mean**	**SE**
*E. antarcticus*	External	6.07	1.25	3.34	0.77	26.75	6.70	5.06	0.84
	Internal	6.92	1.22	3.97	0.73	21.29	4.47	3.67	0.60
*P. murrayi*	External	11.18	1.57	8.82	1.40	16.80	2.91	3.17	0.46
	Internal	13.34	1.65	10.76	1.36	18.77	2.33	3.53	0.31
Tardigrada	External	17.51	4.62	13.08	3.81	34.33	7.12	5.41	0.83
	Internal	18.82	3.81	14.67	2.75	26.29	5.80	4.67	0.78

**SI Table 4:** Differences in eukaryotic community composition of A. all microbiomes, B. external microbiomes, and C. internal microbiomes using a PERMANOVA. Comparisons included microinvertebrate host (*E. antarcticus* or *P. murrayi* or Tardigrada), microbiome type (external or internal), mat type (black or orange), stream (Canada or Bowles Creek or Delta or Von Guerard), and their interactions. The abbreviation “n.a.” is used to depict when a term was not included in the model.

	A. External and Internal Microbiomes		B. External Microbiomes		C. Internal Microbiomes	
	P	R^2^	P	R^2^	P	R^2^
Host	**<0.00**	**0.05**	**<0.00**	**0.09**	**<0.00**	**0.09**
Microbiome	**<0.00**	**0.02**	n.a.	n.a.	n.a.	n.a.
Mat	0.53	<0.00	**0.04**	**0.03**	0.34	0.02
Stream	**0.09**	**0.03**	**<0.00**	**0.09**	0.11	0.06
Host:Microbiome	**<0.00**	**0.03**	n.a.	n.a.	n.a.	n.a.
Host:Mat	0.67	0.02	**0.01**	**0.06**	0.13	0.04
Microbiome Type:Mat	**0.04**	**0.01**	n.a.	n.a.	n.a.	n.a.
Host:Microbiome Type:Mat	**<0.00**	**0.02**	n.a.	n.a.	n.a.	n.a.

Factors explaining the largest variation (R^2^) of each model are underlined and statistically significant differences are highlighted in bold.

**SI Table 5:** **Differences in relative abundance of selected taxa (Cyanobacteria, Bacteroidota, Proteobacteria, Actinobacteriota, *Flavobacterium***, ***Larkinella*****, Fungi, and Metazoa) using a linear mixed model. Comparisons included microinvertebrate host (*E. antarcticus* or *P. murrayi* or Tardigrada), microbiome type (external or internal), mat type (black or orange), stream (Canada or Bowles Creek or Delta or Von Guerard), and their interactions**.

	Cyanobacteria		Bacteroidota		Proteobacteria		Actinobacteriota	
	F	P	F	P	F	P	F	P
Host	**3.85**	**0.03**	**9.06**	**<0.00**	**13.71**	**<0.00**	**5.17**	**0.01**
Microbiome	**6.84**	**0.01**	0.76	0.39	2.38	0.13	0.36	0.55
Mat	0.02	0.88	1.13	0.30	1.21	0.28	0.62	0.44
Stream	**4.92**	**0.01**	**2.74**	**0.07**	0.28	0.84	**3.24**	**0.05**
Host: Microbiome	1.85	0.17	**4.87**	**0.01**	**3.98**	**0.03**	0.23	0.80
Host:Mat	**3.09**	**0.06**	1.88	0.17	**3.26**	**0.05**	0.15	0.86
Microbiome:Mat	0.75	0.39	0.00	0.97	<0.00	0.99	0.26	0.61
Host:Microbiome:Mat	1.93	0.16	0.32	0.73	0.31	0.73	0.10	0.90
	***Flavobacterium***		***Larkinella***		**Fungi**		**Metazoa**	
	**F**	**P**	**F**	**P**	**F**	**P**	**F**	**P**
Host	**6.83**	**<0.00**	**27.80**	**<0.00**	**13.10**	**<0.00**	**7.56**	**<0.00**
Microbiome	**3.44**	**0.07**	**4.73**	**0.03**	2.23	0.14	1.15	0.28
Mat	0.16	0.69	0.00	0.95	0.24	0.63	0.15	0.70
Stream	0.40	0.76	1.59	0.23	**4.01**	**0.02**	0.69	0.57
Host: Microbiome	**3.98**	**0.03**	**6.57**	**0.00**	0.41	0.67	**4.07**	**0.02**
Host:Mat	0.94	0.40	0.17	0.84	0.27	0.77	0.30	0.74
Microbiome:Mat	0.02	0.88	0.08	0.78	0.10	0.75	1.97	0.16
Host:Microbiome:Mat	**3.01**	**0.06**	0.50	0.61	2.04	0.14	4.64	0.01

**SI Table 6:** Means and standard error of the mean (SE) for relative abundance of selected taxa (Cyanobacteria, Bacteroidota, Proteobacteria, Actinobacteriota, *Flavobacterium*, *Larkinella*, Fungi, Metazoa), within external and internal microbiomes. Abundances are reported as a proportion of the entire community (i.e., 0-1).

		Cyanobacteria		Bacteroidota		Proteobacteria		Actinobacteriota	
		Proportion	SE	Proportion	SE	Proportion	SE	Proportion	SE
*E. antarcticus*	External	0.03	0.01	0.75	0.06	0.19	0.08	0.00	0.01
	Internal	0.02	0.01	0.09	0.08	0.81	0.10	0.01	0.00
*P. murrayi*	External	0.22	0.06	0.37	0.05	0.29	0.03	0.02	0.01
	Internal	0.06	0.02	0.64	0.06	0.20	0.04	0.03	0.01
Tardigrada	External	0.04	0.03	0.47	0.07	0.33	0.05	0.01	0.00
	Internal	0.01	0.02	0.62	0.05	0.29	0.03	0.01	0.00
		***Flavobacterium***		***Larkinella***		**Fungi**		**Metazoans**	
		**Proportion**	**SE**	**Proportion**	**SE**	**Proportion**	**SE**	**Proportion**	**SE**
*E. antarcticus*	External	0.72	0.05	0.00	0.00	0.10	0.03	0.64	0.13
	Internal	0.02	0.01	0.00	0.00	0.16	0.07	0.17	0.08
*P. murrayi*	External	0.06	0.01	0.21	0.05	0.53	0.07	0.37	0.06
	Internal	0.02	0.00	0.51	0.06	0.86	0.04	0.04	0.01
Tardigrada	External	0.07	0.03	0.00	0.00	0.37	0.09	0.01	0.00
	Internal	0.13	0.04	0.00	0.00	0.74	0.04	0.04	0.02
